# Model-Based Clustering with Measurement or Estimation Errors

**DOI:** 10.3390/genes11020185

**Published:** 2020-02-10

**Authors:** Wanli Zhang, Yanming Di

**Affiliations:** Department of Statistics, Oregon State University, Corvallis, OR 97330, USA; zhang_wan_li@lilly.com

**Keywords:** gaussian finite mixture model, clustering analysis, uncertainty, expectation-maximization algorithm, classification boundary, gene expression, RNA-seq

## Abstract

Model-based clustering with finite mixture models has become a widely used clustering method. One of the recent implementations is MCLUST. When objects to be clustered are summary statistics, such as regression coefficient estimates, they are naturally associated with estimation errors, whose covariance matrices can often be calculated exactly or approximated using asymptotic theory. This article proposes an extension to Gaussian finite mixture modeling—called MCLUST-ME—that properly accounts for the estimation errors. More specifically, we assume that the distribution of each observation consists of an underlying true component distribution and an independent measurement error distribution. Under this assumption, each unique value of estimation error covariance corresponds to its own classification boundary, which consequently results in a different grouping from MCLUST. Through simulation and application to an RNA-Seq data set, we discovered that under certain circumstances, explicitly, modeling estimation errors, improves clustering performance or provides new insights into the data, compared with when errors are simply ignored, whereas the degree of improvement depends on factors such as the distribution of error covariance matrices.

## 1. Introduction

Model-based clustering [1,2] is one of the most commonly used clustering methods. The authors of [3] introduced the methodology of clustering objects through analyzing a mixture of distributions. The main assumption is that objects within a class share a common distribution in their characteristics, whereas objects from a different class will follow a different distribution. The entire population will then follow a mixture of distributions, and the purpose of clustering would be to take such a mixture and analyze it into simple components and estimate the “probabilities of membership”, that is, the probabilities that each observation belongs to each cluster.

One of the most recent implementations of model-based clustering is MCLUST [4,5,6], in which each observation is assumed to follow a finite mixture of multivariate Gaussian distributions. MCLUST describes cluster geometries (shape, volume, and orientation) by reparameterizing component covariance matrices [7], and formulates different models by imposing constraints on each geometric feature. The expectation-maximization (EM) algorithm [8,9] is used for maximum likelihood estimation, and the Bayesian information criterion (BIC) [10,11] is used for selection of optimal model(s).

In most cases, observations to be clustered are assumed to have been precisely measured, whereas there are situations where this assumption is clearly not feasible. This article proposes an extension to Gaussian mixture modeling that properly accounts for measurement or estimation errors in the special case when the error distributions are either known or can be estimated, as well as introduces the clustering algorithm built upon it, which we named MCLUST-ME. The real data example that motivated our study is where we apply clustering algorithm to coefficients from gene-wise regression analysis of an RNA-seq data set (see Section 3.3 for details). For each gene, five of the fitted regression coefficients correspond to log fold changes in mean expression levels between two groups of *Arabidopsis* plants at five time points after treatment. In such a case, we can reasonably approximate the error covariances of the regression coefficients by inverting the observed information matrix. In general, whenever one applies clustering analysis to a set of summary statistics, it is often possible to approximate the distribution of their estimation errors with, for instance, Gaussian distributions. In this paper, we describe how the estimation/measurement errors, with known or estimated error covariances, can be incorporated into the model-based clustering framework. An obvious alternative strategy in practice is to ignore the individual estimation/measurement errors. We will use simulations and the real data example to understand in what circumstances explicitly modeling the estimation errors will improve the clustering results, and to what degree.

In Section 3.3, we will compare the results of applying the MCLUST method and our new MCLUST-ME method to cluster the log fold changes of 1000 randomly selected genes from the RNA-seq data set mentioned above at two of the time points where the gene expressions were most active. Here, we briefly summarize the input data structure for the clustering analysis and the highlights of the results. Columns 2 and 3 of Table 1 list the estimated log fold changes and their standard errors for 15 representative genes at the two time points being analyzed: these are from the regression analysis applied to each row (gene) of the RNA-seq data set. (The standard errors are the square roots of the corresponding diagonal entries of the error covariances.) In particular, we included 10 genes that are classified differently by the MCLUST and the MCLUST-ME methods. We note that there is sizable variation among the standard errors of the log fold changes. When MCLUST was used to cluster the log fold changes, the estimation errors will be ignored: As long as two genes have the same log fold changes at the two time points, they will always belong to the same cluster. However, we understand that, in this context, a moderate log fold change with a high estimation error is less significant than the same log fold change with low estimation error: this would be obvious if we were to perform a hypothesis test for differential expression (DE), but existing clustering methods such as MCLUST cannot readily incorporate such information into a clustering analysis. The MCLUST-ME method we propose in this paper aims to incorporate information about the estimation errors into the clustering analysis. One distinctive feature of the new MCLUST-ME method is that two points with similar log fold changes may not belong to the same cluster: it also depends on the error covariances of the log fold changes. We note that when the 1000 genes were classified into two clusters by MCLUST and MCLUST-ME, the two clusters for this data set roughly correspond to a “DE” cluster and a “non-DE” cluster. Columns 4 and 5 of Table 1 list the probabilities to the “non-DE” cluster estimated by MCLUST and by MCLUST-ME. We see that the genes that were classified into the “non-DE” cluster by MCLUST-ME, but to the “DE” cluster by MCLUST tend to be genes having moderate log fold changes, but relatively large error covariances. We do not have the ground truth for this data set, but the results from the new MCLUST-ME method alert us that not all log fold changes are created equal.

The organization of the rest of this article is as follows. Section 2 briefly reviews the MCLUST method and then introduces our extension, MCLUST-ME. In particular, Section 2.6 investigates decision boundaries of the two methods for two-group clustering. Section 3.1 and Section 3.2 give simulation settings and results on comparing MCLUST-ME with MCLUST in terms classification accuracy and uncertainty. Section 3.3 gives an example where we cluster a real-life data set using both methods. Finally, conclusions and perspectives for future work are addressed in Section 4.

## 2. Materials and Methods

### 2.1. Review of MCLUST Model

**Finite mixture model**    Let $f_{1}\left(y;\Theta_{1}\right),f_{2}\left(y;\Theta_{2}\right),\ldots ,f_{G}\left(y;\Theta_{G}\right)$ be *G* probability distributions defined on the *d*-dimensional random vector y, and a mixture of the *G* distributions is formed by taking proportions $\left\{\tau_{k}\right\}$ of the population from components $\left\{f_{k}\right\}$, with probability density given by

(1) $$f\left(y;\Theta \right)=\sum_{k=1}^{G}\tau_{k}f_{k}\left(y;\Theta_{k}\right),$$

where $\Theta =(\Theta_{1},\ldots ,\Theta_{G})$ are model parameters.

**Component density**    The MCLUST model assumes that the distribution of each y is a mixture of multivariate normal distributions. Under the MCLUST model, the component density of y in group *k* is

(2) $$f_{k}\left(y;\mu_{k},\Sigma_{k}\right)=\frac{\exp\left\{-\frac{1}{2}{\left(y-\mu_{k}\right)}^{T}\Sigma_{k}^{-1}\left(y-\mu_{k}\right)\right\}}{\sqrt{\det[2\pi \Sigma_{k}]}},$$

In other words,

(3) $$\begin{array}{c} y|k∼N_{d}\left(\mu_{k},\Sigma_{k}\right). \end{array}$$

The (marginal) probability density of y is given by

(4) $$f\left(y\right)=\sum_{k=1}^{G}\tau_{k}f_{k}\left(y;\mu_{k},\Sigma_{k}\right).$$

**Likelihood function**    Suppose a sample of *n* independent and identically distributed (iid) random vectors $y=(y_{1},\ldots ,y_{n})$ is drawn from the mixture. The (observed) log likelihood of the sample is then

(5) $$l_{O}\left(\Theta ;y\right)=\sum_{i=1}^{n}\log f\left(y_{i}\right)=\sum_{i=1}^{n}\log\sum_{k=1}^{G}\tau_{k}f_{k}\left(y_{i};\mu_{k},\Sigma_{k}\right),$$

where $\Theta =(\tau_{1},\ldots ,\tau_{G};\mu_{1},\ldots ,\mu_{G};\Sigma_{1},\ldots ,\Sigma_{G})$ are the model parameters.

### 2.2. MCLUST-ME Model

We extend the MCLUST model by associating each data point with an error term and assumes that the covariance matrix of each error term is either known or can be estimated.

**Component density**    Given that y belongs to component *k*, the MCLUST-ME models assumes that there exists a latent variable w, representing its “truth” part, and ϵ, representing its “error” part, such that

(6) $$\begin{array}{c} \left\{\begin{array}{c} y=w+\epsilon ,\\ w|k∼N_{d}\left(\mu_{k},\Sigma_{k}\right),\\ \epsilon ∼N_{d}\left(0,\Lambda \right), \end{array}\right. \end{array}$$

where w and ϵ are independent. $\mu_{k}$ and $\Sigma_{k}$ are unknown mean and covariance parameters (same as in the MCLUST model), and Λ is the known error covariance matrix associated with y. The distribution of y being in component *k* is then

(7) $$\begin{array}{c} y|k∼N_{d}\left(\mu_{k},\Sigma_{k}+\Lambda \right), \end{array}$$

with density function

(8) $$g_{k}\left(y;\mu_{k},\Sigma_{k},\Lambda \right)=\frac{\exp\left\{-\frac{1}{2}{\left(y-\mu_{k}\right)}^{T}{\left(\Sigma_{k}+\Lambda \right)}^{-1}\left(y-\mu_{k}\right)\right\}}{\sqrt{\det[2\pi \left(\Sigma_{k}+\Lambda \right)]}},$$

and the (marginal) probability density of y is given by

(9) $$g\left(y\right)=\sum_{k=1}^{G}\tau_{k}g_{k}\left(y;\mu_{k},\Sigma_{k},\Lambda \right).$$

**Likelihood function**    Suppose a sample of *n* iid random vectors $y=(y_{1},\ldots ,y_{n})$ is drawn from the mixture, where each $y_{i}$ is associated with known error covariance matrix $\Lambda_{i}$. The (observed) log likelihood of the sample is then

(10) $$l_{O}\left(\Theta ;y\right)=\sum_{i=1}^{n}\log g\left(y_{i}\right)=\sum_{i=1}^{n}\log\sum_{k=1}^{G}\tau_{k}g_{k}\left(y_{i};\mu_{k},\Sigma_{k},\Lambda_{i}\right),$$

where $\Theta =(\tau_{1},\ldots ,\tau_{G};\mu_{1},\ldots ,\mu_{G};\Sigma_{1},\ldots ,\Sigma_{G})$ are the model parameters.

In summary, the MCLUST-ME and MCLUST models have the same set of model parameters for the normal components and the mixing proportions. The key difference is that under the MCLUST-ME model, the measurement or observation errors of the observations are explicitly modeled, and observations are each associated with a given error covariance matrix.

### 2.3. Expectation-Maximization (EM) Algorithm

In the original MCLUST method, the EM algorithm is used to estimate the unknown parameters and compute the membership probabilities. In this subsection, we will first review the EM algorithm under the general MCLUST framework, and then highlight the differences in implementation between the MCLUST method and the MCLUST-ME method.

**Complete data log likelihood**    Given observations $\left(y_{1},\ldots ,y_{n}\right)$, suppose that each $y_{i}$ is associated with one of *G* states. Then, there exists unobserved indicator vectors $\left\{z_{i}=\left(z_{i1},\ldots ,z_{iG}\right)\right\}$ where $z_{i}\overset{\mathrm{iid}}{∼}{\mathrm{Mult}}_{G}\left(1,\tau \right)$ with $\tau =(\tau_{1},\ldots \tau_{G})$. The complete data then consists of $x_{i}=\left(y_{i},z_{i}\right)$. Assuming that the conditional probability density of $y_{i}$ given $z_{i}$ is $\prod_{k=1}^{G}f_{k}{\left(y_{i};\mu_{k},\Sigma_{k},\Lambda_{i}\right)}^{z_{ik}}$, the complete data log likelihood can be derived as follows,

(11) $$\begin{array}{c} \begin{array}{cc} l_{C} & =\log\prod_{i=1}^{n}f\left(y_{i},z_{i}\right)\\ \; & =\log\prod_{i=1}^{n}f\left(y_{i}|z_{i}\right)f\left(z_{i}\right)\\ \; & =\log\prod_{i=1}^{n}\left[\prod_{k=1}^{G}f_{k}{\left(y_{i};\mu_{k},\Sigma_{k},\Lambda_{i}\right)}^{z_{ik}}\right]\left[\prod_{k=1}^{G}\tau_{k}^{z_{ik}}\right]\\ \; & =\log\prod_{i=1}^{n}\prod_{k=1}^{G}{\left[\tau_{k}f_{k}\left(y_{i};\mu_{k},\Sigma_{k},\Lambda_{i}\right)\right]}^{z_{ik}}\\ \; & =\sum_{i=1}^{n}\sum_{k=1}^{G}z_{ik}\log\left[\tau_{k}f_{k}\left(y_{i};\mu_{k},\Sigma_{k},\Lambda_{i}\right)\right]. \end{array} \end{array}$$

**EM iterations**    The EM algorithm consists of iterations of an *M step* and an *E step*, as described below.

- *M step*: Given current estimates of $\left\{z_{ik}\right\}$, maximize the complete-data log-likelihood $l_{C}$ with respect to $\left(\tau_{k},\mu_{k},\Sigma_{k}\right)$.
- *E step*: Given estimates $\left({\hat{\tau }}_{k},{\hat{\mu }}_{k},{\hat{\Sigma }}_{k}\right)$ from last *M step*, for all i=1,…,n and k=1,…,G, compute the membership probabilities
  (12) $${\hat{z}}_{ik}=\frac{{\hat{\tau }}_{k}f_{k}\left(y_{i};{\hat{\mu }}_{k},{\hat{\Sigma }}_{k},\Lambda_{i}\right)}{\sum_{j=1}^{G}{\hat{\tau }}_{j}f_{j}\left(y_{i};{\hat{\mu }}_{j},{\hat{\Sigma }}_{j},\Lambda_{i}\right)}.$$

The two steps alternate until the increment in $l_{O}$ is small enough. Upon convergence, a membership probability matrix is produced and each observation is assigned to the most probable cluster, that is,

(13) $$\begin{array}{c} \mathrm{membership}\;\mathrm{of}\;y_{i}={\mathrm{argmax}}_{k}\left\{{\hat{z}}_{ik}\right\}, \end{array}$$

and the classification uncertainty for $y_{i}$ is defined as

(14) $$1-\max_{k}\left\{{\hat{z}}_{ik}\right\}.$$

In two-group clustering, the classification uncertainty cannot exceed 0.5 (otherwise the point is incorrectly assigned).

For MCLUST, the component density $f_{k}$ is defined in  (2), and for MCLUST-ME, $f_{k}$ is substituted by $g_{k}$ in (8).

**M-step implementation details**    For likelihood maximization in the *M step*, a closed-form solution always exists for ${\hat{\tau }}_{k}$, k=1,⋯,G (see [12] for more details):

(15) $${\hat{\tau }}_{k}=\frac{1}{n}\sum_{i=1}^{n}z_{ik}$$

We can derive the estimation equations for $\mu_{k}$ and $\Sigma_{k}$ (k=1,⋯,G) by taking the partial derivatives of $l_{C}$ with respect to $\mu_{k}$ and $\Sigma_{k}$ and setting the derivatives to 0. For MCLUST-ME (see [13] for a summary of useful matrix calculus formulas, in particular (11.7) and (11.8)):

(16) $$\frac{\partial l_{C}}{\partial \mu_{k}}=\sum_{i=1}^{n}z_{ik}{\left(\Sigma_{k}+\Lambda_{i}\right)}^{-1}\left(y_{i}-\mu_{k}\right)=0$$

and

(17) $$\begin{array}{cc} \; & \frac{\partial l_{C}}{\partial \Sigma_{k}}=\frac{1}{2}\sum_{i=1}^{n}z_{ik}{\left(\Sigma_{k}+\Lambda_{i}\right)}^{-1}\left(y_{i}-\mu_{k}\right){\left(y_{i}-\mu_{k}\right)}^{T}{\left(\Sigma_{k}+\Lambda_{i}\right)}^{-1}-\frac{1}{2}\sum_{i=1}^{n}z_{ik}{\left(\Sigma_{k}+\Lambda_{i}\right)}^{-1}=0. \end{array}$$

For estimation equations under the MCLUST model, one set all the $\Lambda_{i}$’s to 0 in the above two equations. Note that under MCLUST, if there is no constraint on $\Sigma_{k}$, there are closed-form solutions for $\mu_{k}$ and $\Sigma_{k}$:

(18) $${\hat{\mu }}_{k}=\frac{\sum_{i=1}^{n}z_{ik}y_{i}}{\sum_{i=1}^{n}z_{ik}}$$

and

(19) $${\hat{\Sigma }}_{k}=\frac{\sum_{i=1}^{n}z_{ik}\left(y_{i}-{\hat{\mu }}_{k}\right){\left(y_{i}-{\hat{\mu }}_{k}\right)}^{T}}{\sum_{i=1}^{n}z_{ik}}.$$

For MCLUST-ME, each $y_{i}$ corresponds to a different $\Lambda_{i}$. One can see that, in general, there is no closed-form solution for $\left(\mu_{k},\Sigma_{k}\right)$. In our implementation of the MCLUST-ME M step, we solve $\mu_{k}$ from (16),

(20) $${\hat{\mu }}_{k}={\left[\sum_{i=1}^{n}z_{ik}{\left(\Sigma_{k}+\Lambda_{i}\right)}^{-1}\right]}^{-1}\sum_{i=1}^{n}z_{ik}{\left(\Sigma_{k}+\Lambda_{i}\right)}^{-1}y_{i},$$

and plug it into (17), and then use the limited-memory BFGS, a quasi-Newton method in R (function optim [14]), to obtain an optimal solution for $\Sigma_{k}$ numerically. We obtain ${\hat{\mu }}_{k}$ by substituting the resulting ${\hat{\Sigma }}_{k}$ into (20).

The complexity of the EM algorithm for the MCLUST-ME increase with the number of clusters, the number of parameters (which is determined by the dimension of the data), and the number of observations. It is much slower than the original MCLUST algorithm due to the fact we have to use a numerical optimization routine to find the maximum likelihood estimate (MLE) of $\mu_{k}$’s and $\Sigma_{k}$’s in the M step. (See Conclusion and Discussion for a brief summary of running time of MCLUST-ME on the real data example.)

### 2.4. Initial Values

Owing to its iterative nature, the EM algorithm can start with either an *E step* or an *M step*. In the context of model-based clustering, initiation with the *M step* takes advantage of the availability of other existing clustering methods, in the sense that, given a data set, we can acquire their initial memberships by first clustering the data with other methods. MCLUST adopts model-based agglomerative hierarchical clustering [7,15] to generate initial memberships. Model-based hierarchical clustering aims at maximizing the *classification likelihood* instead of (5) or (10); at each stage, the maximum-likelihood pair of clusters are merged together. Although the resulting partitions are suboptimal due to its heuristic nature, model-based hierarchical clustering has been shown to often yield reasonable results and is relatively easy to compute [16]. In light of this, we also use model-based hierarchical clustering to obtain initial memberships for MCLUST-ME. For the choice of initial values when starting with *E step* (i.e., initial parameter estimates), see [17] for a nice discussion.

### 2.5. Model Selection

Within MCLUST framework, selection for the number of clusters can be achieved through the use of the Bayesian information criterion (BIC). Given a random sample of *n* independent *d*-vectors $y=(y_{1},\ldots y_{n})$ drawn from (4) and (9) with some value of *G*, the BIC for this *G*-component mixture model is given by:

(21) $$BIC_{G}=2l_{O}\left(\hat{\Theta };y\right)-\nu_{G}\log\left(n\right),$$

where $\hat{\Theta }$ is the MLE for model parameters, $l_{O}$ is the observed likelihood as in (5) or (10), and $\nu_{G}$ is the number of independent parameters to be estimated. In the most simplistic case, we allow the mean and covariance of each component to vary freely—this is the case we will focus on in this paper. Therefore, for a *G*-component mixture model, we have $\nu_{G}=\left(G-1\right)+Gd+Gd\left(d-1\right)/2$. For comparison purpose, in this paper, we will compare MCLUST-ME results to MCLUST results with the same number of components.

### 2.6. Decision Boundaries for Two-Group Clustering

In this subsection, we examine decision boundaries produced by MCLUST and MCLUST-ME for partitioning a sample into G=2 clusters.

#### 2.6.1. MCLUST Boundary

Suppose we would like to separate a *d*-dimensional i.i.d. random sample $S={\left\{y_{i}\right\}}_{i=1}^{N}$ into two clusters with MCLUST. Let $\left({\hat{\tau }}_{k},{\hat{\mu }}_{k},{\hat{\Sigma }}_{k}\right)$ denote MLEs for $\left(\tau_{k},\mu_{k},\Sigma_{k}\right)$ upon convergence. If we assign each point to the more probable cluster, then the two clusters can be expressed as follows.

(22) $$\begin{array}{c} E_{1}=\left\{y_{i}\in S:{\tilde{\tau }}_{1}f_{1}\left(y_{i};{\hat{\mu }}_{1},{\hat{\Sigma }}_{1}\right)-{\tilde{\tau }}_{2}f_{2}\left(y_{i};{\hat{\mu }}_{2},{\hat{\Sigma }}_{2}\right)>0\right\};\;E_{2}=S\backslash E_{1}, \end{array}$$

and the decision boundary separating $E_{1}$ and $E_{2}$ is

(23) $$B=\{t\in R^{d}:{\tilde{\tau }}_{1}f_{1}\left(t;{\hat{\mu }}_{1},{\hat{\Sigma }}_{1}\right)-{\hat{\tau }}_{2}f_{2}\left(t;{\hat{\mu }}_{2},{\hat{\Sigma }}_{2}\right)=0\},$$

where $f_{k}$, k=1,2, is defined in (2). Equivalently, the boundary *B* is the set of all points in $R^{d}$ with classification uncertainty equal to 0.5. Notice that since the solution set *B* does not depend on *i*, a common boundary is shared by *all* observations. When d=2, under the model assumption of MCLUST, the boundary *B* is a straight line when ${\hat{\Sigma }}_{1}={\hat{\Sigma }}_{2}$, and a conic section when ${\hat{\Sigma }}_{1}\neq {\hat{\Sigma }}_{2}$, with its shape and position determined by the values of the MLEs. This can be shown by simplifying the equality in (23) (see [12] for more details).

#### 2.6.2. MCLUST-ME Boundary

Consider the data $S={\left\{y_{i}\right\}}_{i=1}^{N}$ and each $y_{i}$ is associated with known error covariance $\Lambda_{i}$ for all *i*. Suppose our goal is to partition *S* into two clusters. Let $\left({\tilde{\tau }}_{k},{\tilde{\mu }}_{k},{\tilde{\Sigma }}_{k}\right)$ be MLEs from the MCLUST-ME model. If we assign each observation to the more probable cluster, the two clusters can be expressed as follows,

$$\begin{array}{c} E_{1}^{*}=\left\{y_{i}\in S:{\tilde{\tau }}_{1}g_{1}\left(y_{i};{\tilde{\mu }}_{1},{\tilde{\Sigma }}_{1},\Lambda_{i}\right)-{\tilde{\tau }}_{2}g_{2}\left(y_{i};{\tilde{\mu }}_{2},{\tilde{\Sigma }}_{2},\Lambda_{i}\right)>0\right\};\;E_{2}^{*}=S\backslash E_{1}^{*}, \end{array}$$

where $g_{k}$ is defined in (8). The above decision rule (and therefore boundary) of classifying each point $y_{i}$ now depends not only on the values of MLEs, but also on the error covariance matrix, $\Lambda_{i}$, of $y_{i}$. Instead of producing a common boundary for all points in *S*, the MCLUST-ME model specifies an individualized classification boundary for each $y_{i}$ as follows,

$$\begin{array}{c} B^{*}\left(\Lambda_{i}\right)=\left\{t\in R^{d}:{\tilde{\tau }}_{1}g_{1}\left(t;{\tilde{\mu }}_{1},{\tilde{\Sigma }}_{1},\Lambda_{i}\right)-{\tilde{\tau }}_{2}g_{2}\left(t;{\tilde{\mu }}_{2},{\tilde{\Sigma }}_{2},\Lambda_{i}\right)=0\right\}. \end{array}$$

Similar to our argument in Section 2.6.1, when d=2, $B^{*}\left(\Lambda_{i}\right)$ is either a straight line or a conic section.

When $\Lambda_{i}=\Lambda_{j}$ for some i≠j, that is, when two points are associated with the same error covariance, it can be seen that $B^{*}\left(\Lambda_{i}\right)=B^{*}\left(\Lambda_{j}\right)$, meaning that the two points share a common classification boundary. In the special case where $\Lambda_{i}=\Lambda_{j}$∀i≠j, all boundaries $B^{*}\left(\Lambda_{i}\right)$ will coincide with each other.

One consequence of the existence of multiple decision boundaries is that the classification uncertainty of each point will depend on its corresponding value of $\Lambda_{i}$. In MCLUST, points with high uncertainty (≈0.5) are aligned around the single classification boundary, whereas in MCLUST-ME, each highly uncertain point is close to its own boundary. Consequently, as we will see in Section 3.1, our method allows intermixing of points belonging to different clusters, while MCLUST creates clear-cut separation between clusters.

### 2.7. Related Methods

The authors of [18] discussed a clustering method for data with measurement errors. They also assumed that each observation, $y_{i}$, is associated with a known covariance matrix, ${\tilde{\Lambda }}_{i}$, but they assume that this covariance matrix is for the distance *between the observation and the center of a cluster*. Their conceptual model, using our notation, assumes that

(24) $$y_{i}|k∼N_{d}\left(\mu_{k},{\tilde{\Lambda }}_{i}\right)$$

when observation *i* belongs to cluster *k* (under their model, group membership is deterministic, not probabilistic). Comparing (24) to our MCLUST-ME model (6) and (7), we see that their model lacks the “model-based” element—the covariance matrix $\Sigma_{k}$—for each cluster *k*, k=1,⋯,G. In other words, their ${\tilde{\Lambda }}_{i}$ plays the role of our $\Sigma_{k}+\Lambda_{i}$. This is a crucial difference: we understand that in MCLUST and MCLUST-ME models, $\Sigma_{k}$’s are used to capture different shapes, orientations, and scales of the different clusters. Also, although it is reasonable to assume that the error covariances of the measurements ($\Lambda_{i}$ in MCLUST-ME) are known or can be estimated, it is much more difficult to know $\Sigma_{k}+\Lambda_{i}$ (i.e., ${\tilde{\Lambda }}_{i}$), as we do not where the centers of the clusters are before running the clustering algorithm.

The authors of that paper discussed two heuristic algorithms for fitting *G* clusters into observations: hError and kError. Under their model, they need to estimate the $\mu_{k}$’s for all the clusters and the deterministic (or hard) group memberships for each observation. Both algorithms are distance-based, and not based on an EM algorithm. The hError algorithm is a hierarchical clustering algorithm: it iteratively merges two current clusters with the smallest distances. The error covariances ${\tilde{\Lambda }}_{i}$ were incorporated into the distance formula. For each current cluster *k*, let $S_{k}$ be the collection of observations. The center of cluster *k* is estimated by a weighted average of the observations:

(25) $${\hat{\mu }}_{k}={\left(\sum_{i\in S_{k}}{\tilde{\Lambda }}_{i}^{-1}\right)}^{-1}\sum_{i\in S_{k}}{\tilde{\Lambda }}_{i}^{-1}y_{i}$$

with covariance matrix

(26) $$\Psi_{k}=\mathrm{Var}\left({\hat{\mu }}_{k}\right)={\left(\sum_{i\in S_{k}}{\tilde{\Lambda }}_{i}^{-1}\right)}^{-1}.$$

The distance between any two clusters *k* and *l* is defined by

(27) $$d_{kl}={\left({\hat{\mu }}_{k}-{\hat{\mu }}_{l}\right)}^{T}{\left(\Psi_{k}+\Psi_{l}\right)}^{-1}\left({\hat{\mu }}_{k}-{\hat{\mu }}_{l}\right)$$

The kError algorithm is an extension of the *k*-means method. It iterates between two steps: (1) Computing the centers of the clusters using (25). (2) Assigning each point to the closest cluster based on the distance formula

(28) $$d_{ik}={\left(y_{i}-{\hat{\mu }}_{k}\right)}^{T}{\tilde{\Lambda }}_{i}^{-1}\left(y_{i}-{\hat{\mu }}_{k}\right).$$

We implemented the simpler kError algorithm as described above and applied it the real-data example. We summarized our findings in Section 3.3.

The authors of [19] proposed another extension to the *k*-means method that incorporates errors on individual observations. Under their model, each cluster is characterized by a “profile” $\alpha =(\alpha_{1},\cdots ,\alpha_{m})$, where *m* is the dimension of the data. Each observation, $g_{i}=\left(g_{i1},\cdots ,g_{im}\right)$, from this cluster is modeled as

(29) $$g_{ij}=\beta_{i}\alpha_{j}+\gamma_{i}+\epsilon_{ij},\;j=1,\cdots ,m,$$

where $\epsilon_{ij}∼N\left(0,\sigma_{ij}\right)$ with known error variances $\sigma_{ij}$. The distance from an observation $g_{i}$ to a cluster with profile α is defined as

(30) $$\begin{array}{c} \min_{\beta_{i},\gamma_{i}}\sum_{j=1}^{m}{\left[\frac{g_{ij}-\left(\beta_{i}\alpha_{j}+\gamma_{i}\right)}{\sigma_{ij}}\right]}^{2}, \end{array}$$

essentially the weighted sum of squared errors from a weighted least-squares regression of $g_{i}$ on the profile α. The motivation of this distance measure is that it captures both the euclidean distance and the correlation between an observation and a profile. Their version of *k*-means algorithm, CORE, proceeds by iteratively estimating the profile α for each cluster and then assigning each observation $g_{i}$ to the closest cluster according to (30). We note that their distance measure is less useful for low-dimensional data, as a regression line needs to be fitted between each observation and the cluster profile. If we force the slope $\beta_{i}$ to be 0, then we see that their method will be similar to the kError method in [18].

## 3. Results

In our simulations and real-data example, version 5.0.1 of MCLUST was used.

### 3.1. Simulation 1: Clustering Performance

We simulated data from bivariate normal mixture distribution with different parameter settings, and applied both MCLUST-ME and MCLUST to partition the data into two clusters. The purpose of this simulation is twofold: first, to investigate the degree of improvement in clustering performance by incorporating known error distributions, and second, to study how error structure affects clustering result.

#### 3.1.1. Data Generation

The data were generated from a two-component bivariate normal mixture distribution, where each point is either error-free or associated with some known, constant error covariance. The data generation process is as follows.

(1) Generate ${\left\{h_{i}\right\}}_{i=1}^{n}$ i.i.d. from Bernoulli(η). For each *i*, $h_{i}$ will serve as indicator for error, and on average, a proportion η of data points will be associated with error.
(2) Generate ${\left\{z_{i}\right\}}_{i=1}^{n}$ i.i.d. from Bernoulli(τ). Parameter τ will be the mixing proportion.
(3) For i=1,…,n, generate $y_{i}$ from
  $$z_{i}N_{2}\left(\mu_{1},\Sigma_{1}+h_{i}\Lambda \right)+\left(1-z_{i}\right)N_{2}\left(\mu_{2},\Sigma_{2}+h_{i}\Lambda \right).$$

Values of the above parameters are as follows; $\mu_{1}={\left(0,0\right)}^{T}$, $\mu_{2}={\left(8,0\right)}^{T}$, $\Sigma_{1}=64I_{2}$, $\Sigma_{2}=16I_{2}$, n=300, $\tau_{1}=\tau_{2}=0.5$, and $\Lambda =36I_{2}$. As the values of $z_{i}$ provide us with the true memberships of each observation, we are able to use them to evaluate externally the performance of clustering methods in consideration.

#### 3.1.2. Simulation Procedure

The simulation proceeds as follows.

(1) Choose a value for η from {0.1,0.3,0.5,0.7,0.9}.
(2) Randomly select a random seed.
(3) Generate a random sample following Section 3.1.1.
(4) Run MCLUST and MCLUST-ME, fixing G=2. Initiate with true memberships.
(5) Repeat (2)–(4) for 100 different seeds.
(6) Repeat (1)–(5) for each value of η.

The membership for each observation as well as MLEs upon convergence will be recorded.

#### 3.1.3. The Adjusted Rand Index

In this simulation study, as the true memberships of the observations are available, we can externally evaluate the performance of both clustering methods by calculating the Rand index [20]. Given *n* observations and two partitions *R* and *Q* of the data, we can use a contingency table (Table 2) to demonstrate their agreement.

The Rand index (RI) is defined as

$$\begin{array}{c} \mathrm{RI}=\frac{a+d}{a+b+c+d}. \end{array}$$

There are some pitfalls of the Rand index: for two random partitions, the expected value of RI is not equal to zero, and the value of RI tends to one as the number of partitions increases [21]. To overcome these problems, Hubert and Arabie [22] proposed the adjusted Rand index (ARI), which has an expectation of zero. The ARI is defined as

ARI=RI−Expected(RI)1−Expected(RI)=n2(a+d)−[(a+b)(a+c)+(c+d)(b+d)]n22−[(a+b)(a+c)+(c+d)(b+d)].

ARI takes values between −1 and 1, with an ARI of 1 indicating perfect agreement between two partitions (i.e., RI=1), and an ARI of 0 indicating independence between partitions (i.e., RI=Expected(RI)).

Permutation tests can be used to test whether the observed ARI is significantly greater than zero [23]. Although keeping the numbers of partitions and partition sizes the same as the original data, a large number of pairs of partitions are generated at random and ARI is computed for each generated pair. A randomization *p*-value can then be calculated based on the distribution of generated ARI’s. Similarly, permutation *p*-values can be obtained for testing whether paired ARI values originating from two clustering methods are equal or not.

#### 3.1.4. Simulation 1 Results

**Figure 1:**
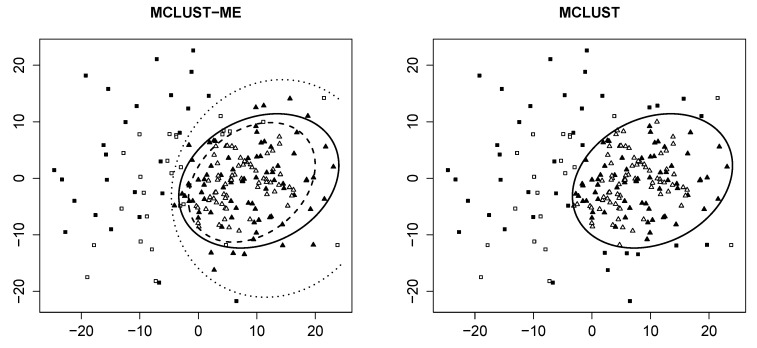
Clustering result of the sample generated with random seed =7 and η=0.5. *Both plots*: empty points represent observations with no measurement errors; solid points represent those generated with error covariance Λ. Clusters are identified by different shapes. *Left*: clustering result produced by MCLUST-ME. Dashed line represents classification boundary for error-free observations; dotted line represents boundary for those with error covariance matrix Λ; solid line represents boundary produced by MCLUST. *Right*: clustering result produced by MCLUST. Solid line is the same as in the left plot.

**Decision boundary** We first visualize the clustering results from both methods, as well as the theoretical decision boundaries stated in Section 2.6. Figure 1 shows groupings of the same data generated with η=0.5 and with random seed 7.

For MCLUST-ME, we identify two distinct decision boundaries: The dotted curve separates points measured *with* errors (solid) into two groups, whereas the dashed curve separates points *without* errors (empty). For MCLUST, one boundary separates all points, regardless of their associated errors. This confirms our findings in Section 2.6.

For this particular simulation, we make two interesting discoveries. First, the two MCLUST-ME boundaries are relatively far apart. Second, none of the three boundaries intersect with each other. As mentioned in Section 2.6.1, the shape and position of these boundaries completely depend upon corresponding values of MLEs, which, in turn, are end results of a procedure of iterative nature (the EM algorithm). We have additional plots similar to Figure 1 for other values of η and other random seeds in [12].

**Figure 2:**
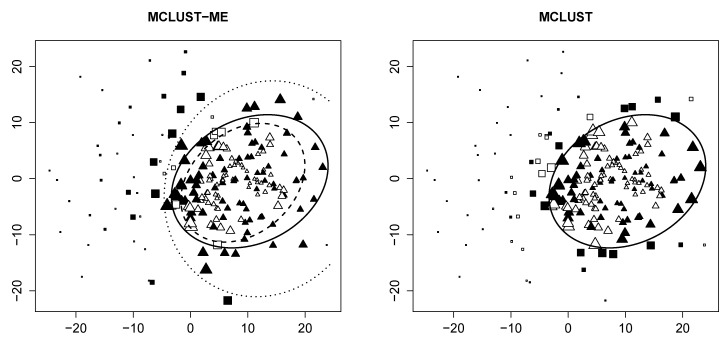
Clustering uncertainty of the sample generated with random seed =7 and η=0.5. Data points of larger size have a higher clustering uncertainty. All other graph attributes are the same as Figure 1.

**Classification uncertainty** In Figure 2, we visualize the classification uncertainty of each point produced by both methods. Observe that for MCLUST, highly uncertain points are found close to the decision boundary, regardless of error. For MCLUST-ME, points with measurement errors (solid) near the outer boundary (dotted) in the overlapping region tend to have high clustering uncertainties. Likewise, error-free points (empty) near the inner boundary (dashed) tend to have high uncertainties. This is consistent with our statement in Section 2.6.2.

**Figure 3:**
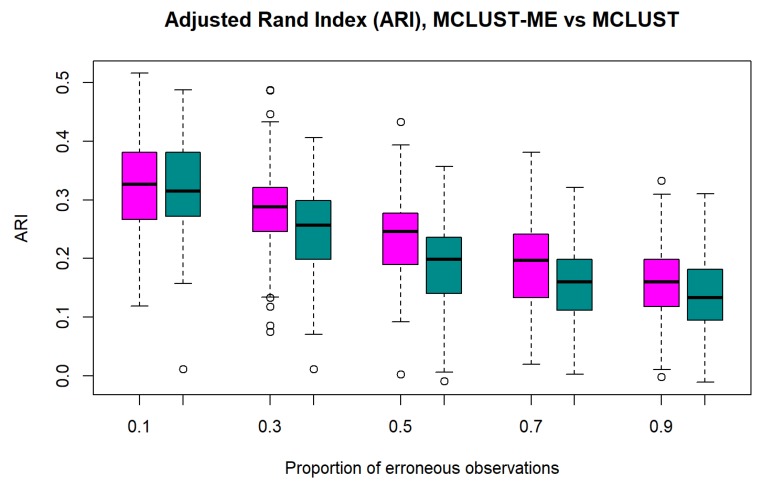
Adjusted Rand indices for MCLUST-ME and MCLUST. Five different proportions of erroneous observations (η) were considered. Magenta: MCLUST-ME; Dark Cyan: MCLUST.

**Accuracy** We first evaluate the performance of MCLUST and MCLUST-ME individually using ARI (between true group labels and predicted labels) as their performance measure. Figure 3 shows that for both methods, clustering accuracy tends to decrease as error proportion η increases. This is intuitively reasonable, because points associated with errors are more easily misclassified due to their high variability, and a larger proportion of such points means a lower overall accuracy.

**Figure 4:**
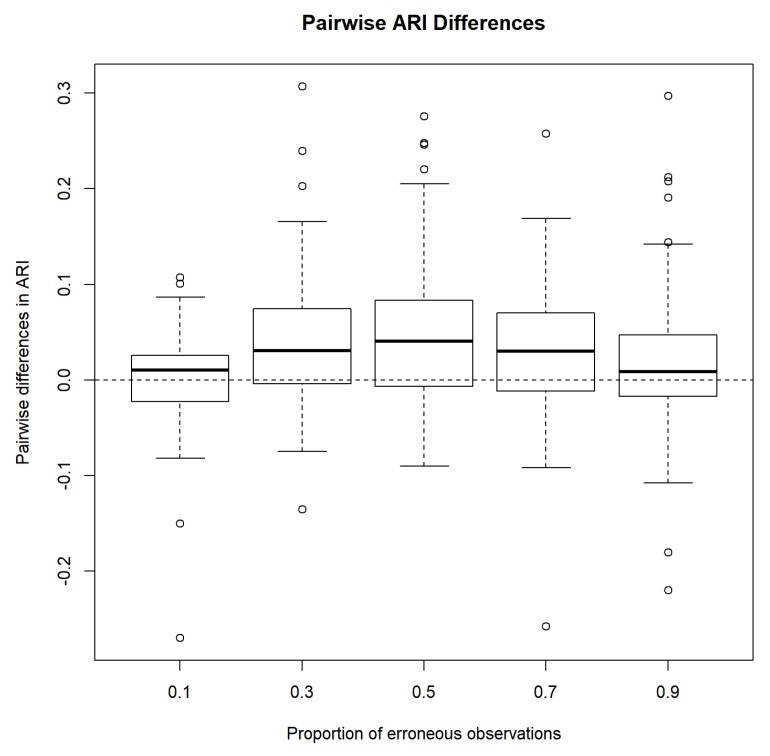
Pairwise difference in adjusted Rand indices between MCLUST-ME and MCLUST. Five different proportions of erroneous observations were considered.

Next, we compare the performances of MCLUST and MCLUST-ME by examining pairwise differences in ARI. Figure 4 shows that on average, MCLUST-ME has a slight advantage in accuracy, and it appears that this advantage is greatest when η=0.5, and becomes smaller as η gets closer to either zero or one. In the latter situation, error covariances will tend to become constant (all equal to $36I_{2}$ as η→1, or 0 as η→0) across all points, meaning that MCLUST-ME will behave more and more like MCLUST, hence diminishing MCLUST-ME’s advantage in accuracy.

Using a permutation test to test the hypotheses $H_{0}:{\mathrm{ARI}}_{MCLUST-ME}={\mathrm{ARI}}_{MCLUST}$ v.s. $H_{1}:{\mathrm{ARI}}_{MCLUST-ME}>{\mathrm{ARI}}_{MCLUST}$, the *p*-values for the five cases are shown in Table 3. With the exception of η=0.1, MCLUST-ME produced a significantly higher ARI than MCLUST.

**Figure 5:**
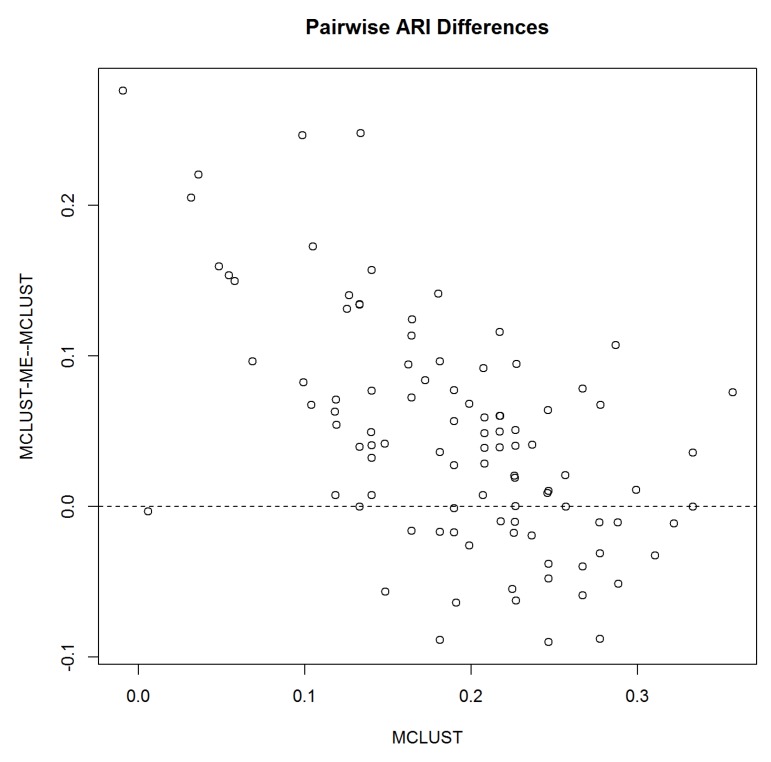
Pairwise difference in accuracy relative to MCLUST accuracy. *X-axis*: MCLUST ARI; *Y-axis*: Pairwise difference between MCLUST-ME and MCLUST ARI values.

Taking a closer look at the pairwise comparison when η=0.5, Figure 5 shows that when MCLUST’s accuracy is low, MCLUST-ME outperforms MCLUST most of the time, and when MCLUST’s accuracy is relatively high, the two methods are less distinguishable on average.

### 3.2. Simulation 2: Clustering Uncertainties and Magnitudes of Error Covariances

In this simulation, our focus is on investigating how clustering uncertainties differ between MCLUST-ME and MCLUST: in particular, we want to see how the magnitudes of error covariances affect the uncertainty estimates. For this purpose, we will let the magnitudes of error covariances vary in a wide range.

#### 3.2.1. Data Generation

The data were generated from a two-component bivariate normal mixture distribution with errors whose magnitudes are uniformly distributed. The data generation process is as follows.

(1) Generate ${\left\{S_{i}\right\}}_{i=1}^{n}$ i.i.d. from Uniform(0,S), where $S_{i}$ denotes the magnitude of error covariance for observation *i*.
(2) Generate ${\left\{z_{i}\right\}}_{i=1}^{n}$ i.i.d. from Bernoulli(τ). Parameter τ will be the mixing proportion.
(3) For i=1,…,n, generate $y_{i}$ from
  $$z_{i}N_{2}\left(\mu_{1},\Sigma_{1}+S_{i}I_{2}\right)+\left(1-z_{i}\right)N_{2}\left(\mu_{2},\Sigma_{2}+S_{i}I_{2}\right),$$
  where $I_{2}$ denotes the 2-dimensional identity matrix.

The parameter values are set as follows; $\mu_{1}={\left(-10,0\right)}^{T}$, $\mu_{2}={\left(10,0\right)}^{T}$, $\Sigma_{1}=\Sigma_{2}=100I_{2}$, n=200, $\tau_{1}=\tau_{2}=0.5$, and S=100. We chose these parameter values so that there will be quite many points near the classification boundary: these points tend to have high classification uncertainties. We want to see, under MCLUST-ME and under MCLUST, how the error magnitudes, $S_{i}$, will affect the estimated classification uncertainties (defined in (14)) of the points.

#### 3.2.2. Simulation Procedure

The simulation proceeds as follows.

(1) Generate a random sample following Section 3.2.1.
(2) Run MCLUST and MCLUST-ME, fixing G=2. Initiate with true memberships.
(3) Record cluster membership probabilities and MLEs for model parameters upon convergence.

#### 3.2.3. Simulation 2 Results

**Figure 6:**
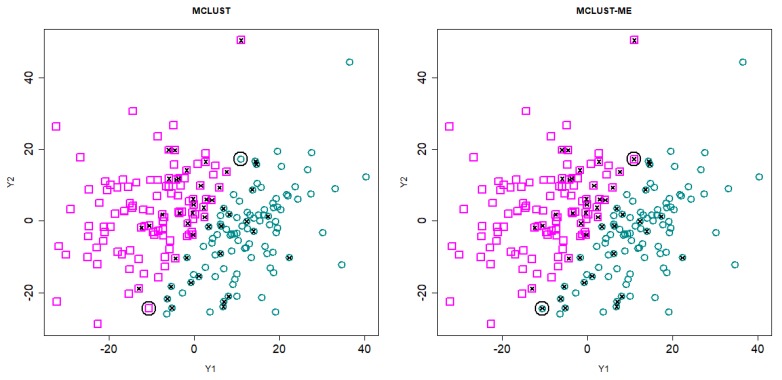
Clustering results for Simulation 2. The clustering results are indicated by different colors and symbols. Points with crosses are misclassified points. The two points that are classified differently by MCLUST-ME and MCLUST are circled in black.

In Figure 6, we show the clustering results from MCLUST-ME and MCLUST (G=2). On this data set, the hard partitioning results do not differ much between the two methods: only two points were classified differently by the two methods (highlighted by black circles).

**Figure 7:**
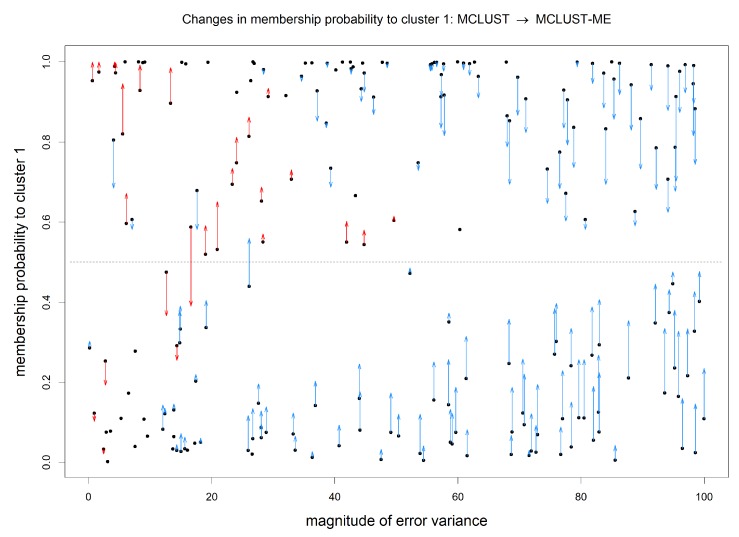
Change in estimated membership probability to cluster 1 from MCLUST to MCLUST-ME, plotted against error magnitude. *X-axis*: magnitude of error covariance, $S_{i}$; *Y-axis*: estimated membership probabilities to cluster 1 by MCLUST (black dots) and by MCLUST-ME (arrowheads). Changes in estimated membership probabilities from MCLUST to MCLUST-ME are highlighted by arrows (no arrow indicates a change less than 0.01). Blue and red arrows indicate an increase and decrease in estimated clustering uncertainty, respectively. With two clusters, the closer the estimated membership probability to 0.5, the higher the classification uncertainty; the classification membership changes when an arrow crosses the horizontal line at 0.5 (the dashed line).

**Figure 8:**
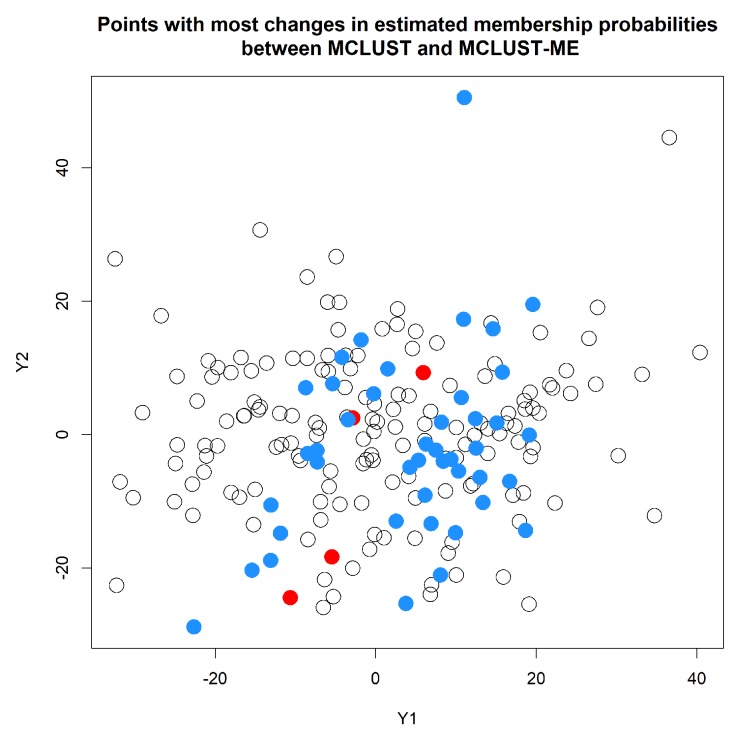
Points with most changes in estimated membership probabilities to cluster 1 from MCLUST to MCLUST-ME. The colored dots correspond to points with a change greater than 0.1 in estimated membership probability to cluster 1. Blue and red colors indicate an increase and decrease in estimated clustering uncertainty, respectively.

Our focus here is on comparing the classification uncertainties estimated under the two methods. For MCLUST, the uncertainty measure for a point depends only on the point location and estimated centers and covariance matrices of the two clusters. Under MCLUST-ME, the uncertainty measure will also depend on the error covariance associated with the point. When two points are at the same location, MCLUST-ME will give higher uncertainty estimate to the point with greater error covariances (see Equation (12)), which is reasonable. In Figure 7, for each observation, we visualize the change in estimated membership probability to cluster 1 between MCLUST and MCLUST-ME with respect to the magnitude of its error covariance($S_{i}$): the closer the membership probability is to 0.5 the higher the classification uncertainty. The points with most changes in estimated membership probabilities are highlighted in Figure 8. Relative to MCLUST, the MCLUST-ME model tends to adjust the classification uncertainties upwards for points with high error covariances and downwards for points with low error covariances. In other words, relative to the MCLUST-ME results, MCLUST tends to overestimate clustering uncertainties for points with low error covariances and underestimate clustering uncertainties for points with high error covariances. This is expected, as MCLUST treats all points as measured with no errors and absorbs all individual measurement/estimation errors into the variance estimates for the two clusters. As a crude approximation, one can think that MCLUST effectively treats each point as having an error covariance matrix close to the average of all true error covariances. However, the up or down changes in membership probabilities (and thus uncertainty estimates) are not a simple function of $S_{i}$, and we do not see a clear-cut boundary between the ups and downs in Figure 7, as the estimates of membership probabilities are also affected by differences in estimates of centers and covariance matrices of the two clusters.

### 3.3. A Real Data Example

#### 3.3.1. Data Description

The data come from an unpublished study on the model plant *Arabidopsis thaliana*. Researchers employed RNA-Seq to create a temporal profiling of *Arabidopsis* transcriptome over a *12h* period, with the aim of investigating plant innate immunity after elicitation of leaf tissue with flg22—a 22-amino-acid epitope of bacterial flagellin. A total of 33 *A. thaliana* Col-0 plants were grown in a controlled environment. Fifteen were treated with flg22, 15 with water, and the other 3 were left untreated. At each of five time points (*10 min, 1 h, 3 h, 6 h, 12 h*), three flg22-treated and three water-treated plants were harvested and prepared for RNA-Seq analysis.

A negative binomial regression model was fitted to each row (i.e., each gene) of the RNA-Seq count data. The regression model was parameterized such that the first five regression coefficients correspond to log fold changes in mean relative expression level between flg22- and water-treated groups at the five time points, which make up the temporal profile of each gene. The regression coefficients were estimated by the MLEs using the R package NBPSeq [24]. Furthermore, based on asymptotic normality of MLE, the covariance matrix of the log fold changes can be estimated by inverting the observed information matrix. For the current study, we will use the estimated regression coefficients and associated variance–covariance matrices for a subset of 1000 randomly selected genes at two of the time points (*1 h* and *3 h*) as input for the clustering analysis, as the gene expressions are most active at these two time points.

#### 3.3.2. Cluster Analysis

**Figure 9:**
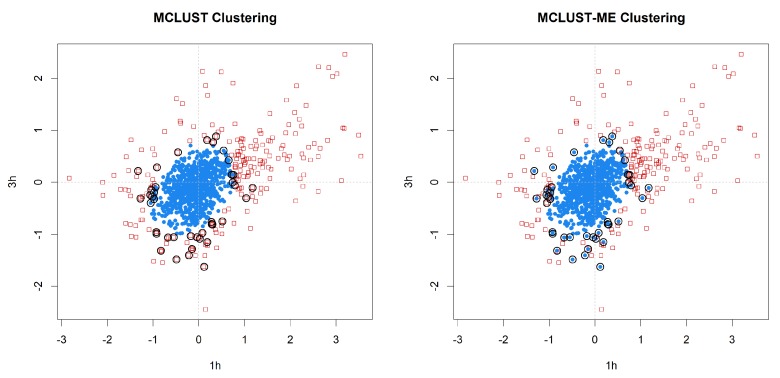
Clustering analysis of the log fold changes of 1000 genes randomly selected from the *Arabidopsis* data set. Two-group clustering of the data with MCLUST-ME and MCLUST, showing log fold changes at 1 h and 3 h. Groups are distinguished by point shapes and colors, and identified as non-DE group (blue circles) and DE group (red squares). Observations classified differently by the two methods are circled in black.

We applied MCLUST-ME and MCLUST to the data. Both methods have their highest BIC values when G=2, 3, or 4. We focus on the G=2 results as it is simple and yet illuminates the key differences between the two methods. In Figure 9, we show the clustering results from the two methods. In this example, both clustering methods show one cluster near the center and another cluster wrapping around it. This makes sense in the context of a gene expression study: the center cluster roughly represent genes that are not differentially expressed (non-DE) at these two time points; the outer cluster roughly represent genes that are differentially expressed (DE). In Figure 9, we see one signature difference between the two clustering methods: MCLUST gives a smooth boundary, whereas in the MCLUST-ME results, the two clusters are interspersed. This is expected from our theoretical analysis earlier and consistent with Simulation 1 results.

**Figure 10:**
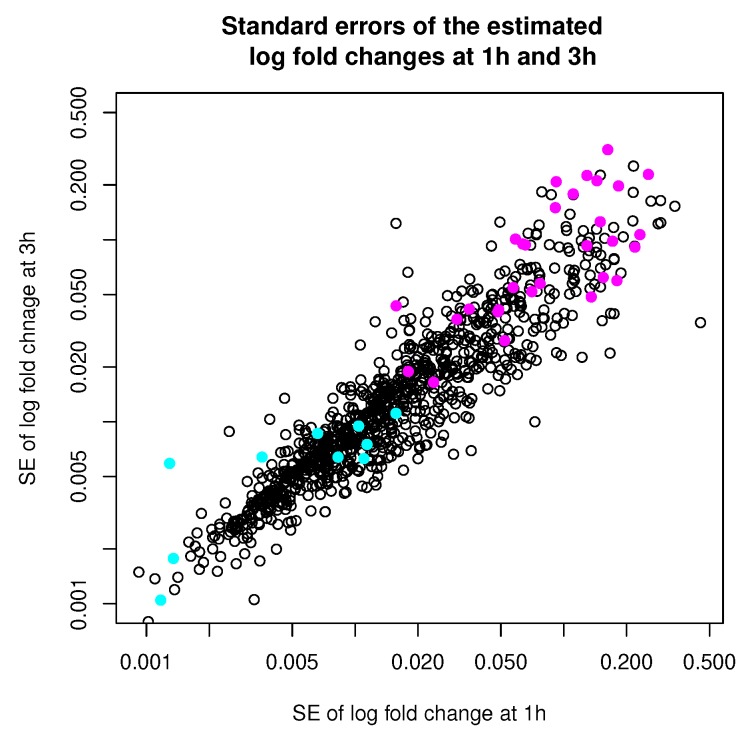
Standard errors of estimated log fold changes at 1 h and 3 h. Observations that are classified differently by MCLUST-ME and MCLUST are highlighted in colors. Magenta: classified as “DE” by MCLUST and as “non-DE” by MCLUST-ME; Cyan: classified as “non-DE” by MCLUST and as “DE” by MCLUST-ME. (Note that the axes are on the log scale.)

Table 4 summarizes the number of points that are classified differently by the two methods. In Figure 10, we show the standard errors (square roots of the diagonal entries of the error covariance) of the log fold changes estimated at 1 h and 3 h, with points classified differently by the two methods highlighted in colors. When we look at the points that are clustered differently by the two methods, we noticed that they tend to be the points either with very low or very high error covariances (relative to the average error covariance). This is expected as we understand that MCLUST absorbs all the individual error covariances into the estimation of the covariances of the two clusters, and thus is effectively using a middle-of-the-pack error covariance to treat each point. Therefore, we expect the differences in clustering results tend to show up among points with either very high or very low error covariances. This observation is also consistent with what we see in Simulation 2.

More interestingly, in this example, we see that the points (genes) that are classified into the “DE” cluster by MCLUST, but into the “non-DE” cluster by MCLUST-ME, tend to have high error covariances. In the MCLUST results, the clustering membership is completely determined by the magnitude of the two regression coefficients, which represent log fold changes between two experimental conditions at the two time points. In MCLUST-ME, membership calculation also considers the estimation uncertainty of the log fold changes. For gene expression data, we know that the uncertainty in log fold change estimation varies greatly (e.g., often depends on the mean expression levels). Although this example is a real data set with no ground truth on each point’s actual group membership, it seems reasonable that points with moderate log fold changes but high error variances should be classified into the non-DE cluster, as MCLUST-ME has done in our example. At the minimum, the MCLUST-ME results warn us that not all points with the same log fold changes are created equal, which is exactly the point we want to highlight in this article. Actually, this example is the data set that motivated us to consider incorporating uncertainty information into the clustering algorithm. In this example, explicitly modeling the error covariances clearly shows a difference.

**Figure 11:**
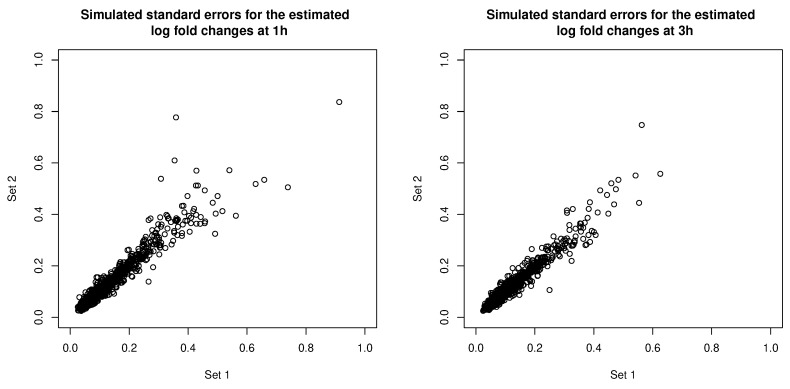
Comparing two sets of simulated standard errors for the estimated log fold changes at 1 h (**left**) and at 3 h (**right**). The standard errors correspond to the square roots of the diagonal entries of the simulated error covariance estimates.

**Figure 12:**
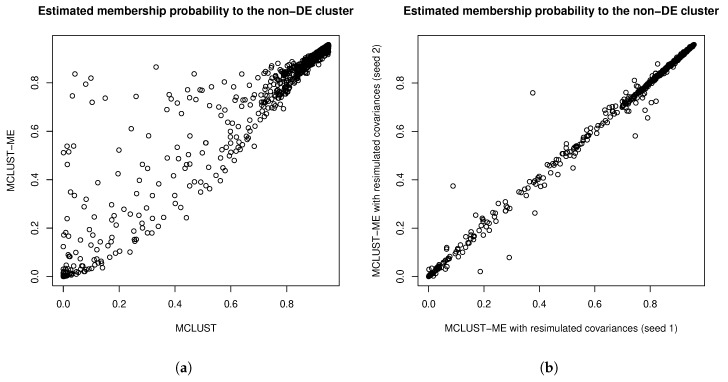
(**a**) Comparing membership probabilities to the “non-DE” cluster estimated by MCLUST-ME and by MCLUST. (**b**) Comparing membership probabilities to the “non-DE” cluster estimated by MCLUST-ME with two sets of simulated covariance estimates. The decision whether or not to model the error covariances will result in drastic changes in the estimated membership probabilities. In comparison, the uncertainties in covariance estimation cause much less changes in the estimated membership probabilities.

The error covariance matrices were estimated, and thus associated with their own estimation errors. To get a sense of the uncertainty associated with estimating the error covariance matrices, we simulated additional sets of error covariance estimates by parametric bootstrapping: simulating copies of the RNA-seq data set based on parameters estimated from the real data set and estimating error covariance matrices from the simulated data sets. In Figure 11, we compare the square roots of the diagonal entries of two sets of simulated error covariance estimates (which correspond to the standard errors of the log fold changes at the two time points). We then tried MCLUST-ME method on the original data set with the two sets of simulated error covariance estimates: eight observations were classified differently due to the differences in error covariance estimates (see Table 5 for a summary). For a closer look, in Figure 12, we show the differences in the estimated membership probabilities (to the non-DE cluster) between the two runs of MCLUST-ME with different simulated error covariance estimates, and these differences were much less than the differences between the original MCLUST-ME and MCLUST results. These results show that the uncertainty in covariance estimation does lead to variation in the clustering results, but the variation is much less as compared to the differences between whether or not to model the estimation errors. In this sense, the MCLUST-ME method is robust to the uncertainty in the covariance estimation to a certain degree.

#### 3.3.3. Comparison to kError

**Figure 13:**
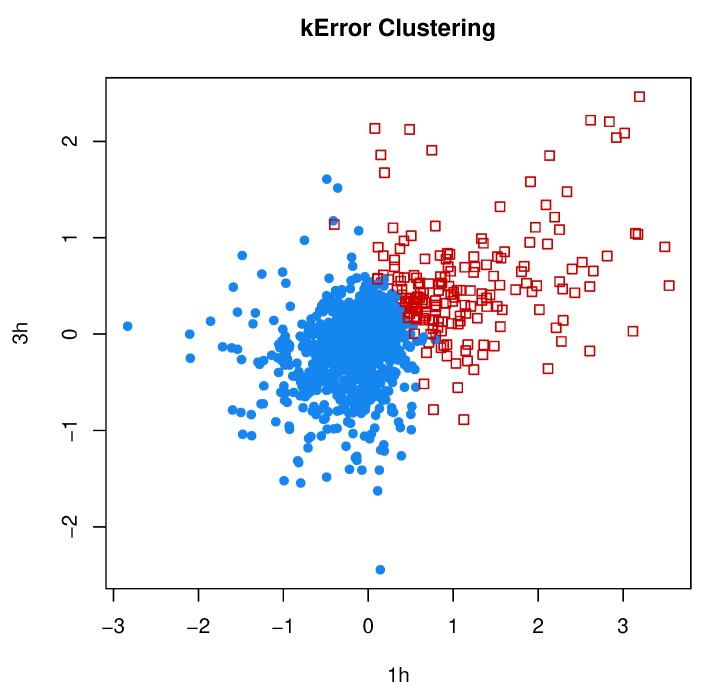
Two-grop clustering results by kError. We applied the kError method to the same RNA-Seq data set that was analyzed by MCLUST and MCLUST-ME. Compare with Figure 9.

In Section 2.7, we reviewed the clustering method by the authors of [18], which models the error covariances of individual observations as in MCLUST-ME, but lacks the model-based components ($N_{d}\left(0,\Sigma_{k}\right)$) for modeling individual clusters. We implemented the kError algorithm according to the description in [18] and applied it to the RNA-Seq data set that we analyzed in the previous subsection, using the estimation error covariances as ${\tilde{\Lambda }}_{i}$ and using the memberships predicted by MCLUST-ME as initial values. The clustering results by kError are shown in Figure 13, which can be compared with the MCLUST and MCLUST-ME results in Figure 9.

For this data set, the two clusters estimated by MCLUST or MCLUST-ME have quite different $\Sigma_{k}$ values: the covariance of the DE cluster is much greater in magnitude than that of the non-DE cluster. The DE cluster is enclosed by the non-DE cluster. Such a structure between the two clusters is difficult for kError method to capture. The way kError split the data sets into two clusters is similar to an ordinary *k*-means method. Interestingly, the two clusters by kError are interspersed without a clean-cut boundary and points with similar values but different covariances can belong to different clusters: This feature is similar to MCLUST-ME.

## 4. Conclusions and Discussion

In this paper, we proposed an extension to model-based clustering approach that accounts for known or estimated error covariances for data observed with uncertainty. The error covariances can often be estimated for data consisting of summary statistics, such as the regression coefficients from a regression analysis. We extended the EM algorithm implemented in MCLUST and implemented our new method MCLUST-ME in R [25].

A distinctive feature of MCLUST-ME is that the classification boundary separating the clusters is not always shared by all observations; instead, each distinct value of error covariance matrix corresponds to a different boundary. Using both simulated and a real data example, we have shown that under certain circumstances, explicitly accounting for estimation error distributions does lead to improved clustering results or new insights, where the degree of improvement depends on the distribution of error covariances.

It is not our intention to claim that MCLUST-ME is universally better than the original MCLUST. We are actually more interested in understanding when it will give different results than MCLUST: in other words, when it is beneficial to explicitly model the measurement error structures when performing clustering analysis. When covariances of estimation errors are roughly constant or small relative to the covariances of the clusters, MCLUST and MCLUST-ME yield highly similar results. We will tend to see meaningful differences when there is significant overlap among clusters (i.e., the difficult cases) and when there is a large variation in the magnitude of error variance.

There are a few natural extensions that can be implemented. For example, in this paper, we focused on the case where the variance–covariance matrices of the clusters are unconstrained (what MCLUST calls “VVV” type). One important feature of the original MCLUST method is that it allows structured constraints on the cluster variance–covariance matrices. Such extension is possible for MCLUST-ME. The main challenge for our current implementation of MCLUST-ME is computational. With MCLUST-ME, each point has its own error covariance matrix, and therefore we no longer have closed-form solutions for estimating the model parameters and have to rely on optimization routines. These factors make MCLUST-ME slower than the MCLUST implementation, but for reasonably-sized low-dimensional data sets, it is still manageable. The running time of the algorithm will depend on the number of clusters (G) and the size and dimension of the observed data. For our real data example, when we classify the 1000 two-dimensional data points into two clusters, it took 19 min. It took 23 h to classify the same data sets into six clusters (on a laptop workstation with an Xeon X3430 processor). To this end, improving the computation routine or exploring approximation methods is a future research topic.

The data and R code for reproducing the results in this paper is available online at https://github.com/diystat/MCLUST-ME-Genes.

## Figures and Tables

**Table 1:** Estimated log fold changes at two time points, associated standard errors, and estimated membership probabilities to the “non-DE” cluster by MCLUST and by MCLUST-ME, for 15 genes selected from the real-data example. Column 2 and 3 are estimated log fold changes at 1 h and 3 h and their standard errors. Column 4 and 5 are estimated membership probabilities to the “non-DE” cluster by MCLUST and by MCLUST-ME. The first 5 rows are randomly selected from 1000 genes that we analyzed. The second 5 rows are selected among the genes that are classified to the “non-DE” cluster by MCLUST, but to the “DE” cluster by MCLUST-ME: the standard errors of the log fold changes tend to be low in this group; the last 5 rows are selected among genes that are classified to the “DE” cluster by MCLUST, but to the “non-DE” cluster by MCLUST-ME: the standard errors of the log fold changes tend to be high in this group.

Gene ID	Log Fold Change (SE) 1 h	Log Fold Change (SE) 3 h	$z_{1}$ MCLUST	$z_{1}$ MCLUST−ME
AT2G42230	−0.277 (0.006)	0.152 (0.006)	0.920	0.921
AT3G56110	0.081 (0.121)	0.228 (0.099)	0.919	0.895
AT1G23330	0.351 (0.018)	−0.209 (0.012)	0.862	0.870
AT5G23060	−0.243 (0.005)	−0.909 (0.005)	0.684	0.751
AT5G06240	−0.680 (0.022)	0.103 (0.012)	0.774	0.764
AT3G20350	−0.952 (0.007)	−0.090 (0.009)	0.562	0.396
AT1G30440	−1.056 (0.010)	−0.398 (0.009)	0.511	0.375
AT1G30490	−0.983 (0.008)	−0.322 (0.006)	0.612	0.480
AT1G23400	−1.017 (0.011)	−0.275 (0.006)	0.547	0.418
AT1G17980	0.734 (0.001)	−0.001 (0.006)	0.524	0.363
AT2G30890	−1.040 (0.150)	−0.142 (0.125)	0.445	0.771
AT5G15160	−0.044 (0.129)	−1.059 (0.225)	0.332	0.866
AT5G45310	−0.221 (0.162)	−1.404 (0.313)	0.042	0.837
AT5G46871	0.373 (0.065)	0.886 (0.094)	0.305	0.581
AT2G22240	0.076 (0.016)	−0.975 (0.043)	0.371	0.690

**Table 2:** 2×2 contingency table for comparing partitions *R* and *Q*.

Partition	*Q*	
*R*	Pair in same group	Pair in different groups
Pair in Same Group	a	b
Pair in Different Groups	c	d

**Table 3:** Permutation *p*-values for comparing MCLUST and MCLUST-ME ARI’s.

η	0.1	0.3	0.5	0.7	0.9
p-**value**	0.256	0	0	0	0.002

**Table 4:** Contingency table for group labels predicted by MCLUST-ME and MCLUST.

		MCLUST-ME	
**MCLUST**		**Non-DE**	**DE**
	Non-DE	775	10
	DE	30	185

**Table 5:** Contingency table for group labels predicted by MCLUST-ME with two sets of simulated error covariance estimates.

		MCLUST-ME Run 1	
**MCLUST-ME Run 2**		**Non-DE**	**DE**
	Non-DE	801	2
	DE	6	191

## Abbreviations

The following abbreviations are used in this manuscript:

ARI	Adjusted Rand index
BIC	Bayesain information criterion
DE	Differentially expressed
EM	Expectation-maximization
MLE	Maximum likelihood estimate(s)
iid	independent and identically distributed
RI	Rand index
